# Rapid and Accurate Assembly of Large DNA Assisted by *In Vitro* Packaging of Bacteriophage

**DOI:** 10.1021/acssynbio.2c00419

**Published:** 2022-11-29

**Authors:** Shingo Nozaki

**Affiliations:** † Department of Life Science, College of Science, Rikkyo University, Tokyo 171-8501, Japan; ‡ Graduate School of Advanced Science and Engineering, Hiroshima University, Hiroshima 739-8527, Japan

**Keywords:** *in vitro* packaging, DNA assembly, phage genome engineering, large plasmid construction

## Abstract

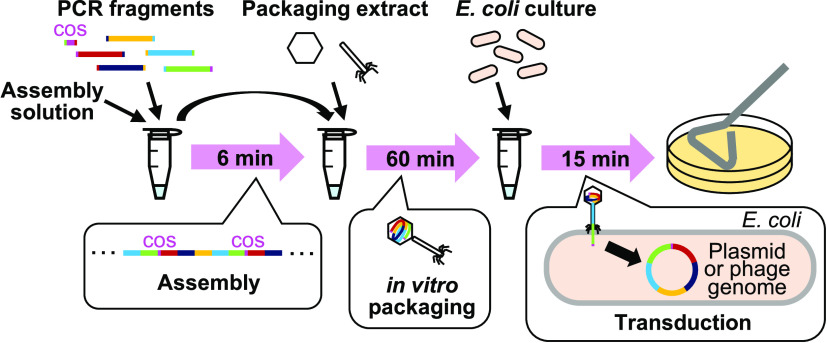

Development of DNA assembly methods made it possible to construct large DNA. However, achieving a large DNA assembly easily, accurately, and at a low cost remains a challenge. This study shows that DNA assembled only by annealing of overlapping single-stranded DNA ends, which are generated by exonuclease treatment, without ligation can be packaged in phage particles and can also be transduced into bacterial cells. Based on this, I developed a simple method to construct long DNA of about 40–50 kb from five to ten PCR fragments using the bacteriophage *in vitro* packaging system. This method, namely, iPac (*in vitro*Packaging-assisted DNA assembly), allowed accurate and rapid construction of large plasmids and phage genomes. This simple method will accelerate research in molecular and synthetic biology, including the construction of gene circuits or the engineering of metabolic pathways.

## Introduction

DNA assembly is one of the fundamental techniques in molecular and synthetic biology. The development of DNA assembly technology has made it possible to assemble various DNA fragments in the desired order. In recent years, there has been an increasing demand for constructing larger and more complex DNA, such as constructing synthetic genomes.^1,2^ However, it is still difficult to achieve large and complex DNA assembly accurately, efficiently, inexpensively, and easily.

DNA cloning into a plasmid vector, namely, two-fragment assembly, was first performed using restriction enzyme and DNA ligase.^3^ This has led to the development of recombinant DNA technology. As a technique for cloning large DNA with restriction enzymes and DNA ligase, cloning into the λ phage vector using *in vitro* packaging of λ phage has been developed.^4−7^ In DNA cloning using the λ phage vector, the λ phage genome itself is used as a vector. The λ phage genome is composed of three domains: left and right arms, which are essential for the lytic cycle including phage proliferation and infection to *Escherichia coli*, and a replaceable region involved in the lysogenic cycle between the left and right arms. In the λ phage vector cloning, the replaceable region is replaced with a DNA fragment to be cloned. The total size including the left and right arms and the cloned DNA should be less than 52 kb, which is the maximum size capable of the λ phage capsid, which can package DNA of 38–52 kb.^8^ The length of the left and right arms is about 29 kb, so a DNA fragment of about 9–23 kb can be cloned with this system. To clone longer DNA, cosmid cloning was developed.^9^ The cosmid cloning uses plasmid vectors containing the cos sequence, which is required for the λ phage packaging system. The size of the cosmid vector is significantly smaller than that of the λ phage vector, and it is possible to clone a DNA fragment of about 30–45 kb. Recently, long regions such as natural product pathways have also been cloned by combining the *in vitro* packaging system of λ phage with the CRISPR-Cas9 system.^10^

In recent years, for the construction of plasmids from multiple DNA fragments, seamless assembly techniques such as SLIC (Sequence and Ligation-Independent Cloning), In-Fusion, Gibson assembly, and Golden Gate assembly are becoming mainstream.^11−15^ In the seamless assembly, the ends of DNA fragments to be assembled are designed to overlap each other. SLIC uses 3′–5′ exonuclease activity of T4 DNA polymerase to generate 5′ overhangs at the ends of insert(s) and a linearized vector. By annealing the overlapping sequences of about 25 bp, insert(s) and the vector are assembled *in vitro*. In the same way, In-Fusion can assemble DNA fragments using vaccinia virus DNA polymerase with shorter DNA overlapping of about 15 bp. Gibson assembly uses thermostable DNA polymerase and DNA ligase in addition to thermolabile T5 5′-3′ DNA exonuclease. After the insert(s) and a linearized vector, DNA is mixed with these enzymes at 50 °C, the exonuclease resects the ends of the DNA fragments to generate 3′ overhangs and is inactivated by the heat. Then, the overlapping ends anneal, the DNA polymerase fills the gaps, and the DNA ligase repairs the nicks. Slightly different from these methods, Golden Gate assembly uses type IIS restriction enzymes. Since the type IIS restriction enzymes cleave DNA sequences distant from recognition sequences,^16^ it is possible to leave short single-stranded DNA overhangs of any sequence at the terminal after the cleavage. The DNA fragments with short overhangs are then ligated by T4 DNA ligase. In these methods, after assembly *in vitro*, the assembled plasmids are generally introduced into *E. coli* cells to purify and amplify the desired plasmids. These methods are used for construction of relatively small plasmids, probably due to the low efficiency of introducing large DNA into *E. coli* cells.

On the other hand, methods for assembling DNA inside cells have also been developed. By utilizing the natural transformation ability of *Bacillus subtilis*, a method to construct large DNA has been developed, in which DNA fragments were added stepwise to the genome of *B. subtilis*.^17−19^*B. subtilis* is not as widespread in many laboratories as *E. coli*, so it has not yet been accessible to many researchers. A method using *Saccharomyces cerevisiae* in constructing large DNA is also attracting attention^20^ because it was used to build the whole genome of *Mycoplasma genitalium* and *Mycoplasma mycoides*.^21−23^ In this method, multiple DNA fragments are simultaneously introduced into yeast cells and assembled by homologous recombination *in vivo*. The DNA assembly by *S. cerevisiae* was also used for construction of phage genomes for phage therapy.^24,25^ However, the growth rate of *S. cerevisiae* is slower than that of *E. coli*, so it takes several days for colonies to appear, which leads to time loss. For a rapid and simple protocol for introducing multiple DNA fragments into *E. coli* and assembling the DNA fragments, IVA (*invivo*assembly) or iVEC (*invivoE. coli*cloning) was also developed.^26−30^ However, it is difficult to assemble large plasmids with this method due to the difficulty in introduction of large or many DNA fragments into *E. coli* cells.

Thus, building large DNA from multiple DNA fragments easily and quickly is still a challenge. This study tackled this challenge by taking advantage of the rapid growth rate of *E. coli* and the ability of a bacteriophage to inject long DNA. The sophisticated ability of phages to introduce DNA into their host cells is attractive. This will make it easier and more inexpensive to introduce long DNA into bacterial cells.

## Results and Discussion

### *In Vitro* Packaging and Transduction of Temporarily Assembled DNA

**Figure 1:**
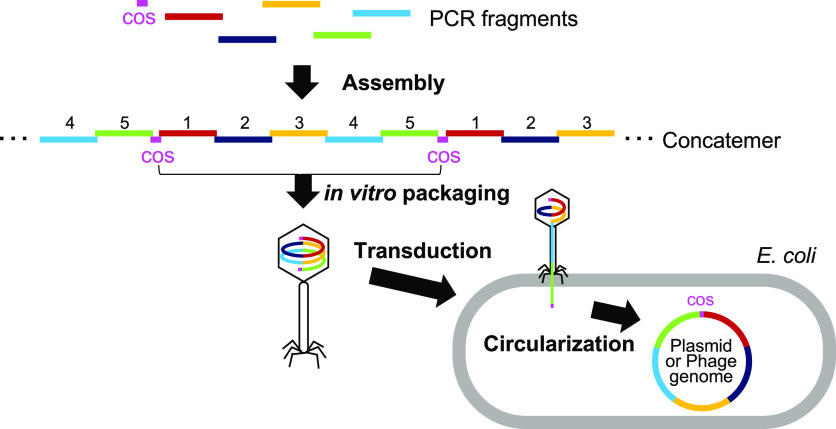
Scheme of iPac (*in vitro*Packaging-assisted DNA assembly). PCR fragments with homologous overlapping ends including the fragments with cos site are assembled by exonuclease treatment and subsequent annealing to form concatemers. The concatemers are packaged into phage particles by *in vitro* packaging. The phage particles are then mixed with *E. coli* cells, and the DNA is transduced into cells. The transduced DNA is circularized at the cos site.

I aimed to establish a simple method to assemble large DNA. Therefore, I attempted to combine simple seamless DNA assembly and long DNA introduction by a bacteriophage (Figure 1).

To verify whether the temporarily assembled DNA can be introduced into *E. coli* cells using the *in vitro* packaging system of λ phage, I first considered a simple DNA assembly method. PCR fragments can be assembled using exonuclease III (Exo III).^31,32^ Exo III has also been shown to contribute to *in vivo* DNA assembly in *E. coli*. Therefore, a simple assembly of PCR fragments was expected to be performed using Exo III. I found that DNA assembly was possible in just a few minutes by adding DNA fragments that overlap 50 bp each with adjacent fragments to the reaction solution containing excess Exo III, with a concentration of around 0.6 U/μL, immediately inactivating the exonuclease at 75 °C and returning the DNA fragments to room temperature for annealing (Figure S1A,B). The incubation on ice after mixing the DNA with assembly solution is not necessary, and increasing the incubation time did not improve assembly efficiency (Figure S1C). The activity of Exo III on the way up to 75 °C is considered sufficient for the assembly. The length of the single-stranded DNA exposed by Exo III treatment cannot be completely controlled. Therefore, it is expected that gaps, flaps, or nicks will occur at the junctions of the DNA fragments after Exo III assembly.

### Construction of λ Phage Genome

The *in vitro* packaging system of λ phage has long been used to introduce DNA into *E. coli* cells.^5,9^ It was reported that the λ phage system allows packaging of heterogeneous DNA with an aberrant structure.^33^ Therefore, I thought it may be possible to package even DNA containing gaps, flaps, or nicks. Then, I examined whether it is possible to package the temporarily assembled DNA that was simply annealed after chewing back with Exo III and to transduce it into *E. coli*.

**Figure 2:**
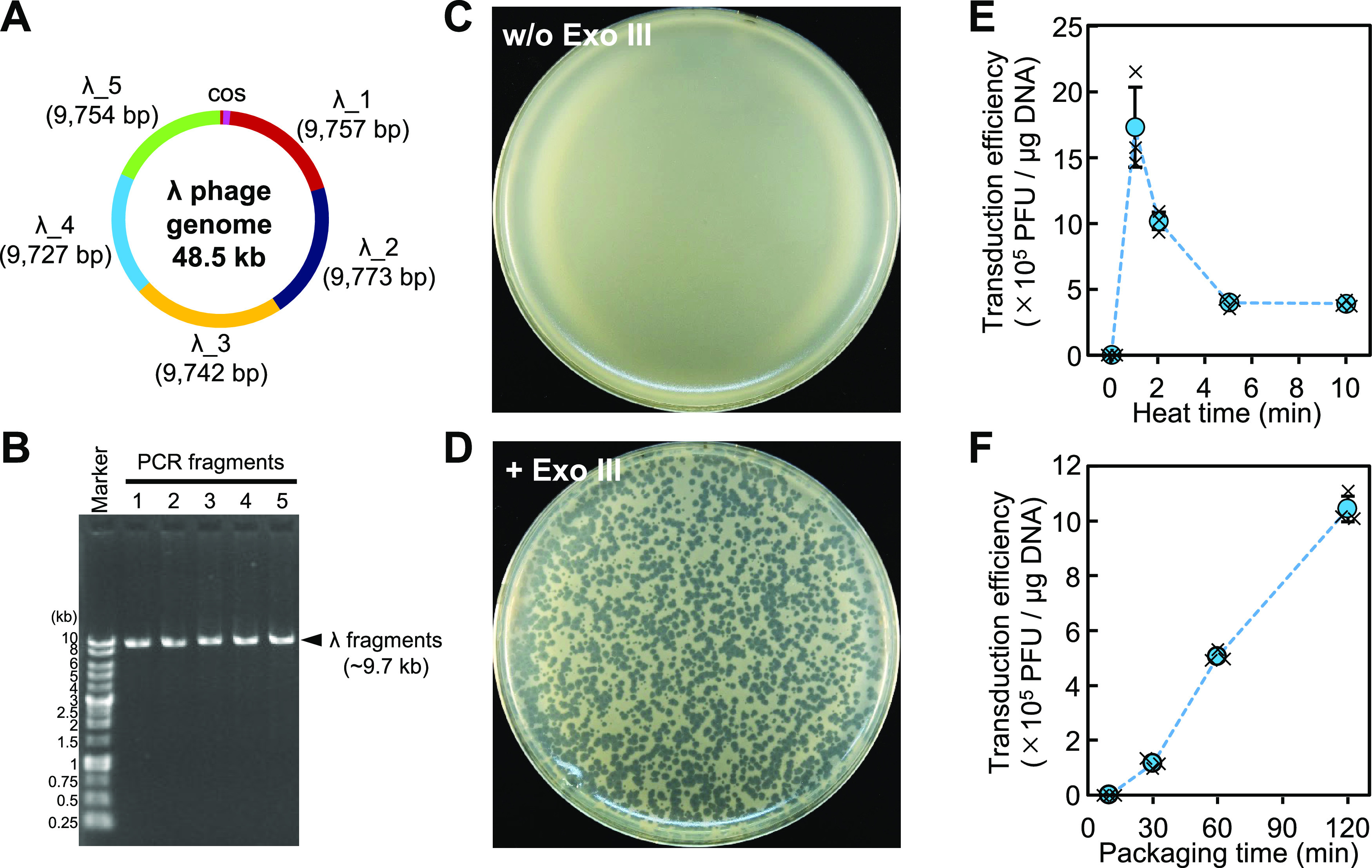
Construction of λ phage by iPac. (A) Design of λ phage genome fragments. λ Phage genome was divided into five fragments of about 9.8 kb (λ_1 to λ_5). The ends of each fragment overlap by 50 bp. The cos site is included in the λ_1 fragment. (B) The five PCR-amplified fragments were analyzed by agarose gel electrophoresis. (C, D) Agar plates containing indicator *E. coli* cells after *in vitro* packaging and transduction of the DNA fragments (C) without or (D) with Exo III assembly. (E) Transduction efficiency according to the heat time after exonuclease treatment. (F) Transduction efficiency according to the time of *in vitro* packaging. The average plaque-forming units of three independent experiments are shown. Error bars and crosses indicate the standard deviations and individual values, respectively.

For this purpose, I attempted to construct a λ phage genome from five PCR fragments of ∼9.7 kb (λ_1 to λ_5), each end of which overlaps with the adjacent fragment by 50 bp (Figure 2A,B). The cos site, which is the packaging site of λ phage, is included in the λ_1 fragment. The PCR fragments were designed to be circular when assembled so that concatemers as packaging substrates could be formed. It is expected that plaques of λ phage will be detected, only when all of these fragments are assembled, packaged into phage capsid, and introduced into *E. coli* cells. No plaque appeared when the PCR fragments without Exo III treatment as negative control were subjected to a packaging reaction and subsequently mixed with the *E. coli* culture (Figure 2C). On the other hand, indeed, many plaques appeared with the Exo III treatment (Figure 2D). Approximately, 3000 plaques appeared using a total of 8.6 ng of PCR fragments with 5 min of heat treatment after Exo III addition and 60 min of packaging time. This corresponds to 3 × 10^5^ PFU/μg DNA.

The number of plaques is expected to increase with improved efficiency in assembly or packaging, so that the plaque number can be a good indicator for better conditions. From this point of view, the most efficient heat time at 75 °C for inactivation during Exo III treatment was 1 min and increasing the heat time reduced the efficiency (Figure 2E). As for the packaging time before mixing with *E. coli* culture, there were almost no plaques in 10 min. Enough plaques of about 5 × 10^5^ PFU/μg DNA appeared with a packaging time of 60 min, and the efficiency was further improved to 1 × 10^6^ PFU/μg DNA by extending it to 120 min (Figure 2F). These results indicate that the temporarily assembled PCR fragments were efficiently packaged by a λ phage packaging system and introduced into *E. coli* cells. Here, I call the DNA assembly method using *in vitro* packaging of bacteriophage as iPac (*in vitro*Packaging-assisted DNA assembly).

Next, I examined whether it was possible to assemble more fragments by iPac. Therefore, I attempted to construct λ phage not only from five DNA fragments, but also from six, eight, and ten DNA fragments. The six, eight, and ten DNA fragments were prepared by dividing one, three, and five DNA fragments, respectively, used in five-fragment assembly into two parts (Figure S2A,B). As a result of assembly of these DNA fragments by iPac, in all cases, λ phage was able to build up and formed plaques (Figure S2C). Up to eight-fragment assembly formed plaques with similar efficiency as a five-fragment assembly (Figure S2C). In the case of ten-fragment assembly, although the efficiency dropped to about 40% of five-fragment assembly, it still allowed assembly with a satisfactory efficiency of 1.7 × 10^5^ PFU/μg DNA.

The assembly procedure prior to *in vitro* packaging will not be essentially limited to the method using Exo III. Various other assembly methods are also expected to work in iPac. In fact, iPac with NEBuilder HiFi DNA assembly kit instead of Exo III assembly was able to construct λ phage from five PCR fragments (Figure S3A). However, the efficiency with this kit was 3% of that with Exo III assembly. This may be because the kit recommends the overlaps of 15–30 bp, which was not suitable for the DNA assembly with the 50 bp overlaps used in this study. It could be improved by optimizing the assembly conditions such as concentration of enzymes.

### Modification of λ Phage Genome by iPac

**Figure 3:**
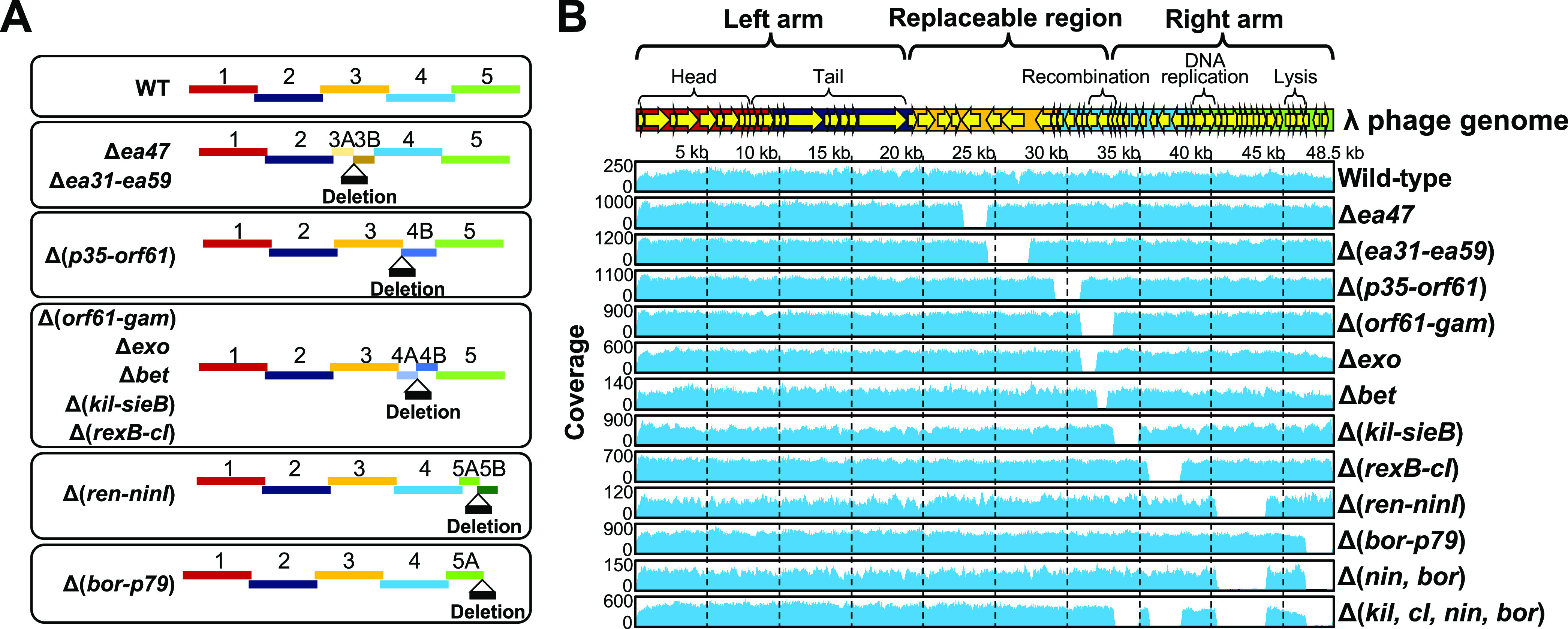
Construction of various deletion mutants of λ phage. (A) Design of fragments for deletion construction. Deletions were introduced between the indicated fragments. The ends of each fragment overlap by 50 bp. (B) NGS analysis of the constructed λ phage genomes. The structure of the λ phage genome is shown at the top. Reads obtained from NGS analysis of the indicated deletion mutants were mapped to the reference λ phage genome sequence (GenBank accession number: NC_001416).

Next, I examined whether λ phage with various deletion mutants could be easily constructed using iPac by changing the PCR fragments used. The PCR fragments were designed so that the ends of adjacent fragments overlap by 50 bp to delete the target regions (Figure 3A). The deletions were introduced into regions of λ phage genome that are not essential for the lytic cycle of λ phage. As a result of NGS analysis of the obtained phage genomes, it was confirmed that the regions of *ea47* (1690 bp), *ea31-ea59* (2938 bp), *p35-orf61* (1843 bp), *orf61-gam* (2278 bp), *exo* (1063 bp), *bet* (750 bp), *kil-sieB* (1754 bp), *rexB-cI* (2359 bp), *ren-ninI* (3496 bp), and *bor-p79* (1877 bp) genes were successfully deleted (Figure 3B). Furthermore, by combining two (*ren-ninI* and *bor-p79*) and four (*kil-sieB*, *rexB-cI*, *ren-ninI*, and *bor-p79*) of these deletions, I succeeded in constructing λ phage with deletions of a total of 5373 and 9486 bp, respectively, by iPac (Figure 3B). In the genome-reduced λ phage, the size of the right arm was reduced from 15.7 to 6.3 kb, and the entire genome size was reduced to 39 kb.

In the cloning using the λ phage vector, the total length of the left and right arms and the DNA fragment to be cloned should be within 52 kb, which can be packaged in the capsid of λ phage, so the shorter the left and right arms of λ phage, the longer DNA fragment can be cloned. Therefore, this genome-reduced λ phage will be used as a λ phage vector that is able to clone up to 27 kb, which is longer than the common λEMBL vector that can be cloned up to 23 kb.^34^

### Construction of Various Phages by iPac

**Figure 4:**
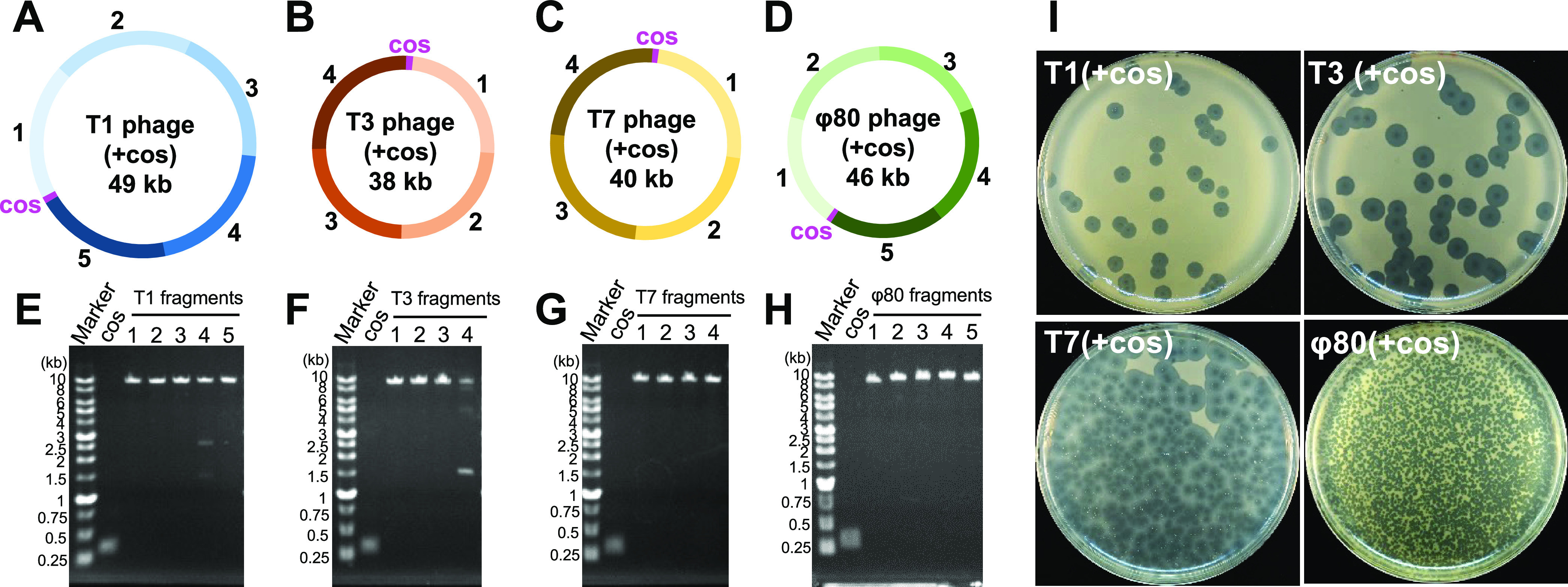
Construction of various phages. (A–D) Design of fragments for construction of (A) T1(+cos), (B) T3(+cos), (C) T7(+cos), and (D) φ80(+cos) phage genomes. The inserted cos sites are indicated as magenta. (E–H) PCR-amplified fragments of (E) T1, (F) T3, (G) T7, and (H) φ80 phage genomes were analyzed by agarose gel electrophoresis. (I) Plaques of the indicated phages that appeared after the transduction into *E. coli*.

So far, the λ phage genomes have been generated using the packaging system of λ phage. Then, can the genomes of other phages be constructed by the λ phage system? To address this question, I selected four other phages, T1, T3, T7, and φ80, to construct. The genome size of these phages is 39–49 kb, which is a packageable size with the λ phage system. I designed the genomes of T1/φ80 or T3/T7 to be constructed from 5 or 4 DNA fragments of about 9–10 kb, respectively, in addition to a 0.3 kb fragment containing the cos site (Figure 4A–D). The DNA fragments were prepared by PCR (Figure 4E–H). The cos site for packaging with λ phage system was placed avoiding regions that might affect transcription: intergenic region between T1p26 (putative tale fiber) and T1p27 (hypothetical protein) gene, between T3 DTR (direct terminal repeat) and T3 promoter phiOL, downstream of AT rich region between T7 promoters A1 and A2, and between φ80 gp35 (hyphothetical protein) and damL (pseudogene) for T1, T3, T7, and φ80, respectively (Figure S2A–D). As a result of introduction of the phage genomes assembled from these DNA fragments into *E. coli* cells by iPac, plaques were obtained in all cases (Figure 4I). The PCR products used for packaging and introduced into *E. coli* cells were 3.9, 3.1, 3.2, and 3.7 ng for phages T1, T3, T7, and φ80, respectively. And the efficiencies were 9600 ± 1200, 18,600 ± 1600, 132,000 ± 1400, and 829,000 ± 105,000 PFU/μg DNA for phages T1, T3, T7, and φ80, respectively (Table 1).

**Table 1:** Number of Plaques Obtained by iPac in the Construction of Various Phages

	number of plaques					
constructed phage	#1	#2	#3	average plaque number ± SD	DNA used (ng)	efficiency (PFU/μg DNA ± SD)
T1(+cos)	33	35	44	37 ± 5	3.9	9600 ± 1200
T3(+cos)	64	53	54	57 ± 5	3.1	18,600 ± 1600
T7(+cos)	426	415	420	420 ± 4	3.2	132,000 ± 1400
φ80(+cos)	3484	2544	3116	3048 ± 387	3.7	829,000 ± 105,000

The phage genomes were recovered from the obtained plaques and analyzed by NGS. For the phages T3, T7, and φ80, the genome was successfully constructed as designed (Figure S4B–D). In the T1 phage, the reads of the inserted cos site were reduced to one-third compared to the other genomic regions (Figure S4A). It is considered that, due to the PCR primer design in T1 phage construction, a homologous sequence of 10 bp was generated on both sides of the cos site, where deletion of the cos site by homologous recombination occurred (Figure S4E). PCR analysis of the cos region in the constructed T1(+cos) phage confirmed that a subpopulation of the phage genomes has the cos site deletion (Figure S4F,G). However, the other region of the T1 phage genome was successfully constructed. Thus, it is possible to construct the phages other than λ phage by iPac using the packaging system of λ phage. NGS analysis also identified zero to four point mutations in each genome (Figure S4A–D). The frequency of mutations was one in 18.3 kb. Since these point mutations were not present at the joints of the fragments, they were considered errors that occurred during PCR, not during the assembly. The use of higher-fidelity DNA polymerases and fewer amplification cycles in PCR would reduce such errors. Thus, iPac has also succeeded in constructing and rebooting the genomes of other phages including lytic phages such as T1, T3, and T7. Because the genome engineering of phages by iPac is simple and rapid, it will be one of the useful options in addition to the conventional phage engineering tools such as for phage therapy.^35,36^

### Construction of a Plasmid by iPac

**Figure 5:**
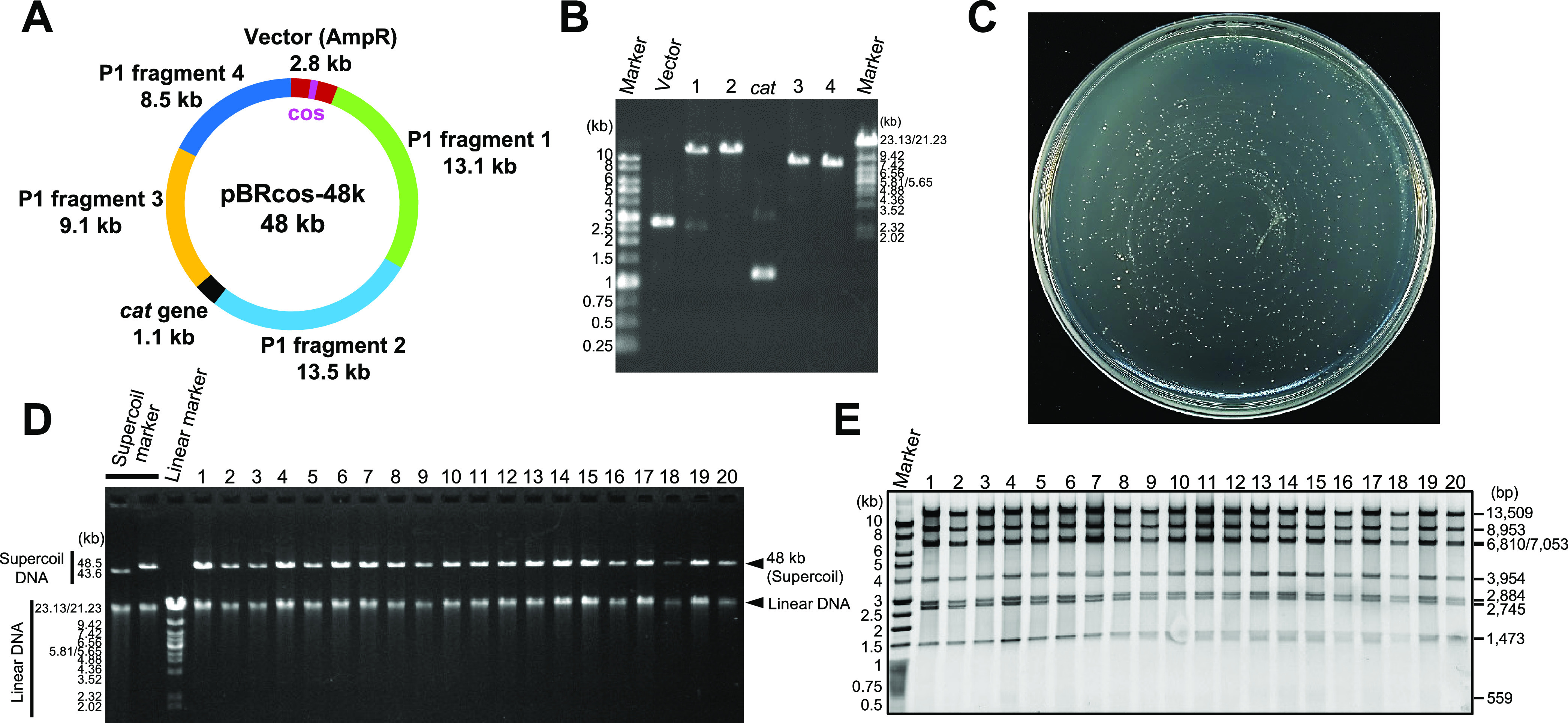
Construction of 48 kb plasmid. (A) Design of 48 kb plasmid; pBR322-cos vector, four fragments from P1 phage genome, and *cat* (chloramphenicol acetyltransferase) gene were used. The ends of adjacent fragments overlap by 50 bp. (B). Verification of PCR-amplified DNA fragments by agarose gel electrophoresis. (C) Ampicillin-resistant colonies that appeared after the transduction into *E. coli* DH5α strain. (D) Agarose gel analysis of the plasmids recovered from 20 randomly selected colonies. Linear DNA is due to contamination of genomic DNA or double-strand breaks of the plasmid DNA. (E) Verification of plasmid construction by a restriction enzyme. The plasmids digested by Nde I were analyzed by agarose gel electrophoresis. The expected sizes of the restriction fragments are indicated on the right.

Next, I examined whether it is also possible to construct a plasmid by iPac. Therefore, I designed a 48 kb plasmid consisting of four fragments from P1 phage genome, *cat* (chloramphenicol acetyltransferase) gene, and a vector fragment containing the ampicillin resistance gene and cos site (Figure 5A). Each fragment was prepared by PCR and was confirmed by agarose gel electrophoresis (Figure 5B). When these PCR fragments were introduced into *E. coli* DH5α strain by iPac and selected only by ampicillin, more than 1000 ampicillin-resistant colonies appeared (Figure 5C). When I recovered the plasmids from 20 randomly selected colonies, surprisingly, all 20 recovered plasmids were of the desired size (Figure 5D). And, all of the colonies were also chloramphenicol-resistant (Figure S5). Furthermore, as a result of confirming the plasmid structure by restriction fragment length analysis, all of the plasmids were correctly constructed (Figure 5E). The average number of colonies in the three independent experiments using 38.2 ng of packaged DNA was 1785. And therefore, the average transformation efficiency was 4.67 ± 0.85 × 10^4^ CFU/μg DNA (Table 2). Thus, iPac could accurately and efficiently construct large plasmids from multiple PCR fragments. Although minor nonspecific bands were visible in the PCR products used (Figure 5B), too short or too long nonspecific products would have been excluded due to the available packaging size limitation of the λ phage head, and thus few false positive colonies would have been generated. However, these nonspecific bands also participated in the assembly reaction and may have reduced the assembly efficiency. Purification of the fragments of interest by extraction from the agarose gel may further increase the efficiency.

**Table 2:** Number of Colonies Obtained by iPac in the Construction of 48 kb Plasmid

number of colonies					
#1	#2	#3	average colony number ± SD	DNA used (ng)	efficiency (CFU/μg DNA)
1821	2161	1372	1785 ± 323	38.2	46,700 ± 8500

### Construction of Smaller Plasmids by iPac

**Figure 6:**
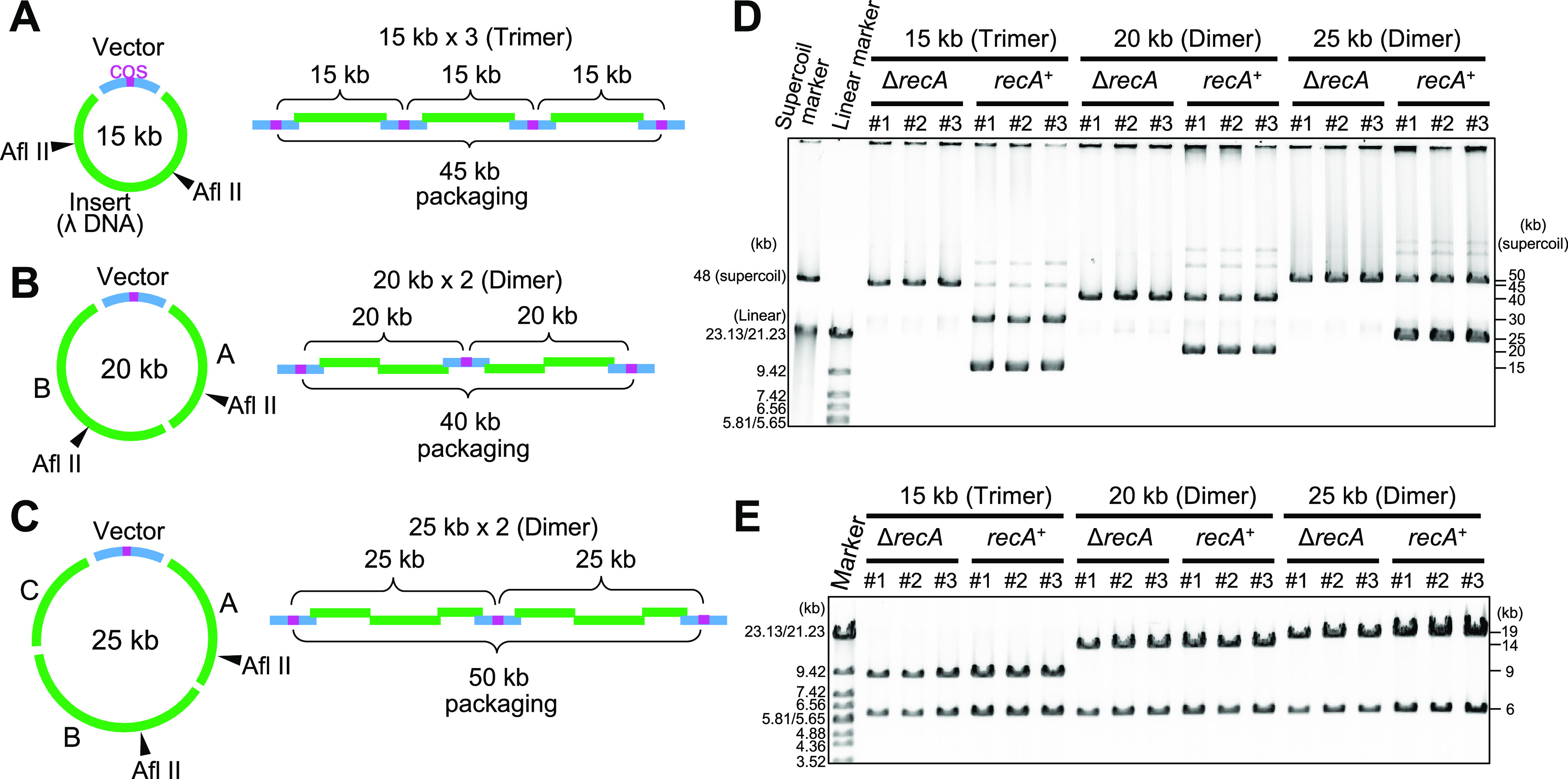
Construction of smaller plasmids by iPac. (A) Design of 15 kb plasmid. The 3 kb vector containing a cos site and the 12 kb fragment of λDNA were prepared. These fragments were assembled to generate concatemers containing the trimer with a packageable size of 45 kb. (B) Design of 20 kb plasmid. The 3 kb vector, and 7 and 10 kb fragments of λDNA were prepared. These fragments were assembled to generate a dimer of 40 kb. (C) Design of 25 kb plasmid. The 3 kb vector, and 7, 10, and 5 kb fragments of λDNA were prepared. These fragments were assembled to generate a dimer of 50 kb. (D). Agarose gel electrophoresis analysis of the plasmids recovered from the *recA*^+^ or Δ*recA* strain of *E. coli* after introduction of the assembled DNA fragments shown in (A), (B), and (C) by iPac. (E) Verification of plasmid construction by a restriction enzyme. The plasmids digested by Afl II were analyzed by agarose gel electrophoresis. The expected sizes of the restriction fragments are indicated on the right.

It has been reported that shorter plasmids as small as 4 kb in size carrying cos site can also be packaged in the λ head in a multimeric manner.^37^ This means that the DNA packaged into the λ head can have multiple cos sites. Inspired by that, I examined to construct plasmids of 15, 20, and 25 kb, which are smaller than the lower limit of the packageable size of about 38 kb. The 15 kb plasmid was designed to be packaged as a trimer of 45 kb, and 20 and 25 kb plasmid as a dimer of 40 and 50 kb, respectively (Figure 6A–C). The DNA fragments were prepared by PCR (Figure S6). These were assembled by iPac and introduced into Δ*recA* and *recA*^+^*E. coli* strains, resulting in the formation of colonies with efficiencies of 3 × 10^4^ – 2 × 10^5^ CFU/μg DNA (Table 3). Each of the three colonies was randomly picked up, and the plasmids were recovered. All of the plasmids recovered from the Δ*recA* strain were confirmed to have a trimer of 45 kb for the 15 kb plasmid and a dimer of 40 and 50 kb for the 20 and 25 kb plasmid, respectively, as designed (Figure 6D). On the other hand, in the case of the *recA*^+^ strain, although dimers, trimers, and tetramers were also detected to be mixed, monomeric plasmids were successfully constructed (Figure 6D). The structure of these plasmids was analyzed by restriction enzyme analysis, and all of the plasmids were found to have the correct structure (Figure 6E). Next, to purify the monomers from the mixture with multimers prepared in *recA*^+^ strain, the 15, 20, or 25 kb plasmids, which are the mixture of monomers and multimers, were introduced into DH5α, a Δ*recA* strain, and the plasmids were recovered. As a result, the monomers of each plasmid were successfully purified, although in the case of 25 kb plasmid, the plasmid from one of the two colonies examined was a dimer (Figure S7). Thus, iPac was able to construct smaller plasmids that are of a packageable size in a multimeric state. It would be also useful for constructing the desired multimers.

**Table 3:** Number of Colonies Obtained by iPac in the Construction of 15, 20, and 25 kb Plasmids

		number of colonies					
plasmid size	host strain	#1	#2	#3	average colony number ± SD	DNA used (ng)	efficiency (CFU/μg DNA)
15 kb	Δ*recA*	5121	4651	4842	4871 ± 193	24.1	201,721 ± 7992
	*recA*^+^	4663	4254	4062	4326 ± 250	24.1	179,152 ± 10,379
20 kb	Δ*recA*	953	1169	1156	1093 ± 99	32.0	34,161 ± 3092
	*recA*^+^	1329	1059	1441	1276 ± 160	32.0	39,903 ± 5013
25 kb	Δ*recA*	5317	5478	5139	5311 ± 138	39.8	133,471 ± 3479
	*recA*^+^	5565	5457	5016	5346 ± 237	39.8	134,342 ± 5968

### Advantages of iPac and Future Challenges

When constructing a large circular plasmid from multiple DNA fragments *in vitro*, the concentration of the DNA fragments should be lowered to increase the efficiency of self-circularization by joining intramolecular ends, although the efficiency of assembly between each DNA fragment decreases. On the other hand, the efficiency of the assembly rises by increasing the concentration of the DNA fragments. But in this case, the concatemers produced by intermolecular end joining are more likely to form instead of circular plasmids. In the iPac system, the concatemer is the substrate for packaging, and the circularization occurs inside the cells after transduction. Therefore, this conflicting problem is solved, enabling highly efficient assembly. In addition, iPac does not require special equipment such as an electroporator or high-performance competent cells, which are generally required for the introduction of large DNA into *E. coli*. The transduction in iPac is completed simply by mixing the *E. coli* cells prepared from the overnight culture and is performed at a low cost.

The problem with the current iPac system is that the DNA size that can be packaged in the capsid of λ phage is limited to about 38–52 kb. Using the *in vitro* packaging system of phages with larger genomes, such as P1 and T4 phages with genome sizes of 94 and 169 kb, respectively, it will be possible to construct DNA larger than the packaging limit of λ phage system. Furthermore, using the packaging system of phages that infect other bacteria, the iPac system may be applicable to other bacteria. On the contrary, limiting the size of the packaged DNA can also be beneficial. Generally, in the assembly of multiple DNA fragments, it is inevitable that the wrong assembly results in the construction of the wrong size plasmid. In the iPac system, the assembled DNA that is too small or too large to complete packaging into the phage capsid is excluded during the packaging process. It is considered that this size elimination mechanism made it possible to construct the plasmid with extremely high accuracy (Figure 5D,E). In this study, up to 10 DNA fragments were assembled by iPac. There is room for further research as to whether it can be applied to the assembly of more fragments. By Golden Gate assembly, 40 kb genome of T7 phage was constructed from 52 DNA fragments.^38^ Combining iPac with Golden Gate assembly may improve the efficiency of multifragment construction. Here, I demonstrated the construction of the phage genomes and the large plasmids using portions of phage genomes as DNA fragments. The GC contents of the DNA used were around 50%. Further studies such as assembly of DNA with higher or lower GC content and integration of metabolic pathway genes will make iPac more practical for applications.

### Accessibility to iPac

To use the iPac system presented here, the packaging extract and the cos sequence are required. Commercially available *in vitro* packaging kits such as LAMBDA INN *in vitro* packaging kit (Nippon Gene) and Gigapack III Gold (Agilent) could be used for iPac (Figure S8). In addition, the packaging extract can also be homemade.^6,39,40^ Homemade packaging extract was also able to perform iPac with a similar efficiency as the commercial one (Figure S8). Cos-less lysogens, SN2099 and SN2100, to prepare homemade packaging extract were deposited to National BioResource Project (NBRP) *E. coli*. In these lysogenic strains, a region from the *SRRz* gene to the cos site has been deleted so that the packaging extract can be prepared from a single strain. The homemade packaging extract will further reduce the cost for iPac. Recently, an *in vitro* transcription/translation system has been put into practical use for phage reconstitution,^41^ which may allow the reconstitution of the packaging extracts not limited to λ phage as well. The cos sequence is required for the packaging using the λ phage system. The cos site is included in some of the commonly used vectors such as the BAC vector, pBeloBAC11, and fosmid vector, pFOS1.^42^ Therefore, plasmid construction by iPac can be readily performed using these vectors without preparing the cos fragment individually.

## Conclusions

I have presented that it is possible to construct large DNA rapidly and accurately using only the materials accessible to many researchers such as *E. coli* and *in vitro* packaging systems. This method will lower the barriers to entry for research using larger DNA.

## Methods

### Bacterial Strains and Medium

*Escherichia coli* strains used for this study are listed in Table 4. SN1171, SN1187, BL21(DE3), and DH5α were used for the recipient strains for transduction. SN1187 was used as the Δ*recA* strain. For homemade packaging extract preparation, SN2099 or SN2100 was used. These strains were constructed by knocking out the region from the *SRRz* gene to the cos site of the λ phage genome integrated into the attλ site of the host strain by the method of Datsenko and Wanner.^43^ LB broth (1% trypton, 0.5% yeast extract, 1% NaCl) (Nacalai Tesque) was used for liquid culture. The agar plates were made by adding 1.5% agar to LB broth. Then, 50 μg/mL ampicillin was added to the medium when antibiotic resistance selection was needed. MgSO_4_ with a final concentration of 10 mM and 0.7% agar were added to LB broth to make soft agar for plaque assay.

**Table 4:** Bacterial Strains

strain	genotype	source or refs
SN1171	F-, *rph-1, ΔhsdR*, Δ*endA*	Nozaki and Niki^26^
SN1187	F-, *rph-1, ΔhsdR, ΔendA*, Δ*recA,*	Nozaki and Niki^26^
SN2099	F-, *rph-1, ΔhsdR*, Δ*endA*, λ+(*cI857*, Δ*SRRz-cos*::*cat*)	this work
SN2100	F-, *rph-1, ΔhsdR*, Δ*endA*, λ+(*cI857*, Δ*SRRz-cos*::*kan*)	this work
DH5α	F-, *deoR, endA1, gyrA96, hsdR17(rK-, mK+), recA1, relA1, supE44, thi-1, Δ(lacZYA- argF)U169, (Phi80lacZ*Δ*M15)*	lab stock
BL21(DE3)	F-, *dcm, ompT, hsdS(rB-mB-), gal, λDE3*	lab stock

### Bacteriophages

Genome of λ phage (*cI857, Sam7*) was purchased from NIPPON GENE. The lambda phage strain (*cI857*) in which *Sam7* mutation was reverted to the wild type was obtained from a plaque that appeared after infection of λ *cI857 Sam7* to *E. coli* SN1171 strain without the amber suppressor mutation. Phages T1, T3, T7, φ80, and P1kc were distributed from the Biological Resource Center, NITE, Japan

### Plasmids

pBR322-cos, a plasmid containing the packaging site of λ phage, was constructed by inserting cos sequence between the replication origin and β-lactamase gene of pBR322^44^ by the iVEC method as described previously.^26^ For amplification of the chloramphenicol acetyltransferase (*cat*) gene, pKD3^43^ was used as a template.

### Preparation of PCR Products

PCR was carried out with KOD One DNA polymerase (TOYOBO) according to the manufacturer’s instructions. The oligonucleotide primers were designed so that the DNA fragments to be assembled overlap each other by 50 bp at the ends. The oligonucleotide primer sequences and combinations of the primers and templates are listed in Tables S1 and S2. The PCR products were purified using the NucleoSpin PCR clean-up kit (Takara).

### *In Vitro* Packaging-Assisted DNA Assembly and Transduction

For preparing the assembly reaction, 2× assembly solution (20 mM Tris-HCl (pH 7.9), 100 mM NaCl, 20 mM MgCl_2_, 10% (w/v) PEG8000, 2 mM dithiothreitol, 1.2 U/μL exonuclease III (Takara)) and an equal amount of 4.8 nM each of DNA fragments were mixed to carry out the assembly reaction. In practice, a 5 μL volume of the reaction (2.5 μL of 2× assembly solution + 2.5 μL of DNA mix) was prepared on ice and incubated at 75 °C for 1 min immediately after the addition of the DNA mix. The combinations of DNA fragments to be assembled for each construct are shown in Table S3. Then, the reaction was incubated at 25 °C for 5 min to allow annealing at exposed single-stranded ends. A portion of assembly reaction (0.5 μL) was mixed with 4 μL of the packaging extract from the *in vitro* packaging kit, LAMBDA INN (NIPPON GENE), and kept for 60–120 min at 25 °C for *in vitro* packaging. As for the packaging extract, Gigapack III Gold (Agilent) or homemade packaging extract prepared from the cos-less lysogenic strain was also used when indicated. For transduction, 1 mL of an overnight culture of *E. coli* cells was collected by centrifugation (5000 g for 1 min). For construction of phages, *E. coli* strain SN1171 was used except for T3 phage construction in which BL21(DE3) was used. For construction of the 48 kb plasmid, DH5α was used. The collected cells were resuspended in 500 μL of 10 mM MgSO_4_. The cell suspension (100 μL) was mixed with 4.5 μL of the *in vitro* packaging reaction and incubated at 25 °C for 15 min. For plaque formation in phage construction, all of the transduced cell suspension or, when counting numerous plaques, 10 μL of the transduced cell suspension and 90 μL of overnight *E. coli* culture were mixed with 2.5 mL of soft agar prewarmed at 50 °C and spread on an LB agar plate. For plasmid construction, the transduced cells were spread on an LB agar plate with 50 μg/mL ampicillin. The agar plates were incubated at 37 °C overnight, and plaques or colonies that emerged on the plates were analyzed.

### NEBuilder HiFi DNA Assembly

Five fragments of the λ phage genome, that overlap 50 bp each with adjacent fragments, were assembled using NEBuilder HiFi DNA Assembly Master Mix (New England Biolabs); 10 μL of DNA fragments with 0.24 pmol of total amount were mixed with 10 μL NEBuilder HiFi DNA Assembly Master Mix on ice and incubated at 50 °C for 60 min as per manufacturer’s instruction. The assembled DNA was used for *in vitro* packaging.

### Next-Generation Sequencing (NGS) Analysis

The libraries from the recovered phage genomes for NGS analysis were prepared with Nextera DNA Flex Library Prep kit (Illumina). The libraries were sequenced using iSeq. 100 system (Illumina). The obtained reads were analyzed by Geneious software (Biomatters Ltd.) with the reference genome sequences of phages λ, T1, T3, T7, and phi80 (GenBank accession numbers: NC_001416, NC_005833, NC_003298, NC_001604, and NC_021190, respectively).

### Plasmid Analysis

Plasmids constructed by iPac were recovered with QIAprep Spin Miniprep kit (Qiagen). The purified plasmids were analyzed by 0.7% agarose gel electrophoresis. The plasmid structure was verified by restriction enzyme treatment. For restriction enzyme digestion, 2.5 μL of purified plasmids were digested with Nde I or Afl II (New England BioLabs) and analyzed by 0.7% agarose gel electrophoresis.

## Supporting Information Available

Sequences of oligo DNA primers used for PCR; primer sets and templates for PCR; combination of PCR fragments for large DNA assembly; DNA assembly conditions by Exo III; and result of NGS analysis of constructed phages (PDF)

## Abbreviations

PCR: polymerase chain reaction
NGS: next-generation sequencing
PEG: polyethylene glycol
PFU: plaque-forming unit
CFU: colony-forming unit
