# Supplementary material for: Rapid and Accurate Assembly of Large DNA Assisted by In Vitro Packaging of Bacteriophage
Source: ACS Synth Biol. 2022 Nov 29;11(12):4113–22. doi: 10.1021/acssynbio.2c00419 (PMC9764419; doi:10.1021/acssynbio.2c00419)
Supplement: Supplementary file 1 — sb2c00419_si_001.pdf [file sb2c00419_si_001.pdf]

Supporting information

Rapid and Accurate Assembly of Large DNA Assisted by *in vitro* Packaging of Bacteriophage

Shingo Nozaki* [1, 2]

[1]Department of Life Science, College of Science, Rikkyo University, Tokyo 171-8501, Japan
[2]Graduate School of Advanced Science and Engineering, Hiroshima University, Hiroshima 739-8527, Japan
*Email: shnozaki@hiroshima-u.ac.jp

Table S1: Oligonucleotide primers used for PCR

**Name**	**Oligonucleotide sequence (5' - 3')**
ON454	CCACGCACGTTGTGATATGT
ON178	GACGGTAATTTCTGCAACC
ON179	CGGTTGTATCCGGTAATGGTGAG
ON180	ATACCCGGGAGTGATTTCC
ON709	GGTCCGGCAGTACAATGGATT
ON182	TAATTGCGGAGACTTTGCGATG
ON183	GTGCGTTCCACTCCTGAAG
ON184	GCGTAACCATCATCGAGATCTG
ON185	AACTTTGCCGGACAGGAGC
ON455	GGACCCGTAAAGTGATAATG
ON970	TGTTGATTGAGTCTCTTTGGTC
ON971	GTAAATCCCGTCTCTCAGGAG
ON972	TTTGTCGGTGTACCTCTCTC
ON973	TGCACCATATTCCTGAACAAC
ON974	GCGTACAGTCATAGATGGTCG
ON975	TGCATGAGAGAATTTGTACCAC
ON976	GTGAATACACGGAGCAATGTC
ON977	AACAAGTCGGCTCCTGTTTAG
ON978	ACCTGCTGATGATCAACTGG
ON979	TATCCCTTTGACGAATACGAG
ON513	<u>CAAAATCCGGTAGTAACTTGCTAAC</u>TGGCGGTGATGTAAACACTA

ON514	GTTCATAGTGTTTACATCACCGCCAGTTAGCAAGTTACTACCGGA
ON515	CCCACACCCAGCATGCATACCTTTCTCCGGTAGTAACTTGCTAAC
ON516	AATTGGTTAGCAAGTTACTACCGGAGAAAGGTATGCATGCTGGGT
ON519	CTGTGTGCTTATGCTTGCCGACATAATTGCGGAGACTTTGCGATG
ON520	AAGTACATCGCAAAGTCTCCGCAATTATGTCGGCAAGCATAAGCACA
ON517	ATGTTTCGTGAAGCCGTCGACGCTTTGTGCTTATGCTTGCCGACA
ON518	TCCCATGTCGGCAAGCATAAGCACAAAGCGTCGACGGCTTCACGA
ON522	CGCAGAGCAGAAGGTGGCAGCATGATGTGCTTATGCTTGCCGACA
ON521	TCCCATGTCGGCAAGCATAAGCACATCATGCTGCCACCTTCTGCT
ON526	ATAAAACGAATGAGTACTGCACTCGAGCAGAAGGTGGCAGCATGAC
ON525	CGGTGTCATGCTGCCACCTTCTGCTCGAGTGCAGTACTCATTCGT
ON645	TAGTTACTTAGATATTGGCCTTGGCTCGTGAAGCCGTCGACGCTT
ON646	TTTATAAGCGTCGACGGCTTCACGAGCCAAGGCCAATATCTAAGT
ON647	GAACTTGGCTTATCCCAGGAATCTGATTGGCCGTAAGTGCGATTC
ON648	ATCCGGAATCGCACTTACGGCCAATCAGATTCCTGGGATAAGCCA
ON610	TGGGGACGCATAATAGCTTCTGTGCGCAGTGTTCTGCGGAAACCT
ON611	ATGTTAGGTTTCCGCAGAACACTGCGCACAGAAGCTATTATGCGT
ON505	CATTTACGAATGTTTGCTGGGTTTCCCACGCACGTTGTGATATGT
ON506	CATCTACATATCACAACGTGCGTGGGAAACCCAGCAAACATTCGT
ON748	TATTACTTCATCTCAATTGCCTTCGCCACGCACGTTGTGATATGT
ON749	ACGTAGACTCATTTCCGAAGGCAATATGTCAGCCAGCTGCTTTTTG
ON750	TCAACAAAAAGCAGCTGGCTGACATATTGCCTTCGGAAATGAGTC
ON751	CTGACCATTCAATCCTCTGC
ON752	GAGCTTTGACACGTTTGAGG
ON753	TAATCAAGACCCATGCAGTC
ON754	GAACTCTTGTCCGCTACGAG
ON755	CCTGTTTCATATCCTCTTCG
ON756	GGGTTCAAATGAGACTGGAG
ON757	GCCGAACTTACTACCATCAAC
ON758	ATCCGGGAGAGATAATCACTC
ON759	CATCTACATATCACAACGTGCGTGGCGAAGGCAATTGAGATGAAG
ON760	TAAAGAGCGACGCTATCTTAAAGACCCACGCACGTTGTGATATGT
ON761	TAACAGTGGGTCTATCGGTCTGGTTCCATTGTTCATTCCACGGAC
ON762	TTTTTGTCCGTGGAATGAACAATGGAACCAGACCGATAGACCCAC
ON763	TTCGCCAAAGTTCATAGTCG

ON764	AAAGCAGCTTGAGAGTAAGG
ON765	ATAAGCGTGCTTCTATCTGG
ON766	TGACTTTACCGTAGCGAAAG
ON767	TGGTGTCCATGAGGTAAATG
ON768	GCAACTGACCATGTATTCTC
ON769	CATCTACATATCACAACGTGCGTGGGTCTTTAAGATAGCGTCGCT
ON770	CTATAGGATACTTACAGCCATCGAGCCACGCACGTTGTGATATGT
ON771	TGGGATGGCTATTCGCCGTGTCCCTCCATTGTTCATTCCACGGAC
ON772	TTTTTGTCCGTGGAATGAACAATGGAGGGACACGGCGAATAGCCA
ON773	ATCTATCAAAGGGGACCTCC
ON774	ACCTGCTGAGTGGATAAAGG
ON775	TCGCATCCAAGTCTGCATAG
ON776	TAGCCTCTAAGCTCATGCTG
ON777	CGTCCGTAGATGAACTTAGG
ON778	AAACGAGTCCGAGAGAAACG
ON779	CATCTACATATCACAACGTGCGTGGCTCGATGGCTGTAAGTATCC
ON780	TTAATGGATTGCTGACCACTTCACCCCACGCACGTTGTGATATGT
ON781	TGGTAACTGCTGGCTAATACCATGGCCATTGTTCATTCCACGGAC
ON782	TTTTTGTCCGTGGAATGAACAATGGCCATGGTATTAGCCAGCAGT
ON783	CGGCAATAACCGTATTTGTC
ON784	CTACGGCTGGAACAAGAAAC
ON785	GATAGCACCATTTGCGATAG
ON786	GCAATGGAGTGTCATTCATC
ON787	CTGATAATCGAAGCCTTGTG
ON788	GTCTCGATTCACTGTTAACG
ON789	TTGAAGTGACCCGGAAATAC
ON790	GGCGATAGACAGTGATAACC
ON791	CATCTACATATCACAACGTGCGTGGGGTGAAGTGGTCAGCAATCC
ON593	GGTCTGACAGTTACCAATGC
ON594	TCGTTCCACTGAGCGTCAGA
ON597	GCATTGGTAACTGTCAGACCCCACGCACGTTGTGATATGT
ON598	TCTGACGCTCAGTGGAACGACCATTGTTCATTCCACGGAC
ON705	GCCGTCCTGATAGGCTTTGATGATCGGCATTGACCCTGAGTGATT
ON706	GACCACTACCGCTCTTTTGTCGTTTGGGCCTCGTGATACGCCTAT
ON707	TAAAAATAGGCGTATCACGAGGCCCAAACGACAAAAGAGCGGTAG

ON651	CATAAGCCGTAATCACCAGG
ON652	AGGATTTATCGGGCCAGTTG
ON653	TAATTCCAGTTTAAAAGGAGGTGAAAACCTCCTTTTATGACATCCAACA
ON550	TGTTGGATGTCATAAAAAGGAGGTTTTCACCTCCTTTTAAACTGGAATTACAT ATGAATATCCTCCTTAG
ON549	GGGCTGGGGAGGCGGCGCTTTGTTGCTAAATGATCTGGTTTAAAATGGATGT GTAGGCTGGAGCTGCTTC
ON654	ATCCATTTTAAACCAGATCATTTAGCAACAAAGCGCCGCCTCCCCAGCCC
ON655	CGGAGTGGAATTCGATTTTG
ON656	TAAGGTATACCCGGAAGGTG
ON708	AGAAAAATCACTCAGGGTCAATGCCGATCATCAAAGCCTATCAGG
ON476	CCCGGCGTTCGGGCTTTTCTGTTATAATTTATTTGGCGGCAACAC
ON477	CATCTACATATCACAACGTGCGTGGATGCCTGGTACTTTGCCAAC
ON456	TCATAGTTGGCAAAGTACCAGGCATCCACGCACGTTGTGATATGT
ON457	GATTTCTTTCACGATCCCGTTTTGTATGTCAGCCAGCTGCTTTTT
ON458	TCAACAAAAAGCAGCTGGCTGACATACAAAACGGGATCGTGAAAG
ON459	CATCCTGTCACCTACTGATTAGCCCGCCATCAAACCACGTCAAAT
ON460	TGATTATTTGACGTGGTTTGATGGCGGGCTAATCAGTAGGTGACAGG
ON461	CAAGCTATCTATGGGGGTGTTTCGCGTAAACGACAACCCCGAAAG
ON462	GCGCGCTTTCGGGGTTGTCGTTTACGCGAAACACCCCCATAGATA
ON463	TTCGGGAGGAGGGGAGGAAGGTAAACAGGCGAAGGCGATAATAGT
ON464	CGTTAACTATTATCGCCTTCGCCTGTTTACCTTCCTCCCCTCCTC
ON465	CAATTTTGTTTCCTCGTGAAGAACCAAAAGCTGAACCCGACACAT
ON736	GACAGCATCGCCAGTCACTAGCCTTATCCGGAGAGGATGA
ON737	AATCACTCAGGGTCAATGCCTTGTCGGTGTACCTCTCTCG
ON738	AATCACTCAGGGTCAATGCCCCTGAAATCTTTACTGCCAT
ON739	AATCACTCAGGGTCAATGCCGTGCCTAGCAAACTCGGAAG
ON740	CGAGAGAGGTACACCGACAAGGCATTGACCCTGAGTGATT
ON741	ATGGCAGTAAAGATTTCAGGGGCATTGACCCTGAGTGATT
ON742	CTTCCGAGTTTGCTAGGCACGGCATTGACCCTGAGTGATT
ON743	TCATCCTCTCCGGATAAGGCTAGTGACTGGCGATGCTGTC
ON968	TATCGTGCGAGACGTTGAAG
ON969	GATGGGTATAGCGACGGAGA

Underlines indicate the additional overhangs.

Table S2: Primer sets and templates used for PCR

DNA fragment	Size (bp)	Forward primer	Reverse primer	Template
λ_1	9757	ON454	ON178	λ cI857 genome
λ_2	9773	ON179	ON180	λ cI857 genome
λ_3	9742	ON709	ON182	λ cI857 genome
λ_4	9727	ON183	ON184	λ cI857 genome
λ_5	9754	ON185	ON455	λ cI857 genome
λ_1A	5438	ON454	ON970	λ cI857 genome
λ_1B	4368	ON971	ON178	λ cI857 genome
λ_2A	5141	ON179	ON972	λ cI857 genome
λ_2B	4682	ON973	ON180	λ cI857 genome
λ_3A	4959	ON709	ON974	λ cI857 genome
λ_3B	4833	ON975	ON182	λ cI857 genome
λ_4A	4975	ON183	ON976	λ cI857 genome
λ_4B	4802	ON977	ON184	λ cI857 genome
λ_5A	4972	ON185	ON978	λ cI857 genome
λ_5B	4832	ON979	ON455	λ cI857 genome
λ_3(Δea47)A	3387	ON181	ON513	λ cI857 genome
λ_3(Δea47)B	4716	ON514	ON182	λ cI857 genome
λ_3(Δea31-ea59)A	5092	ON181	ON515	λ cI857 genome
λ_3(Δea31-ea59)B	1758	ON516	ON182	λ cI857 genome
λ_3(Δp35-orf61)	9766	ON181	ON519	λ cI857 genome
λ_4(Δp35-orf61)B	7860	ON520	ON184	λ cI857 genome
λ_4(Δorf61-gam)A	1939	ON183	ON517	λ cI857 genome
λ_4(Δorf61-gam)B	5560	ON518	ON184	λ cI857 genome
λ_4(Δexo)A	1914	ON183	ON522	λ cI857 genome
λ_4(Δexo)B	6750	ON521	ON184	λ cI857 genome
λ_4(Δbet)A	2998	ON183	ON526	λ cI857 genome
λ_4(Δbet)B	5980	ON525	ON184	λ cI857 genome
λ_4(Δkil-sieB)A	4237	ON183	ON645	λ cI857 genome
λ_4(Δkil-sieB)B	3785	ON646	ON184	λ cI857 genome
λ_4(ΔrexB-cI)A	6450	ON183	ON647	λ cI857 genome
λ_4(ΔrexB-cI)B	968	ON648	ON184	λ cI857 genome
λ_5(Δren-ninI)A	1636	ON185	ON611	λ cI857 genome

λ_5(Δren-ninI)B	4668	ON610	ON455	λ cI857 genome
λ_5(Δbor-p79)A	7852	ON185	ON506	λ cI857 genome
λ_1(Δbor-p79)	9782	ON505	ON178	λ cI857 genome
λ_5(Δnin Δbor)B	2791	ON610	ON455	λ cI857 Δbor-p79 genome
λ_4(Δkil, ΔcI)	4880	ON183	ON647	λ cI857 Δkil-seiB genome
λ_5(ΔcI Δnin Δbor)	5179	ON648	ON455	λ cI857 Δnin Δbor genome
cos(T1)	345	ON748	ON749	λ cI857 genome
T1_1	9819	ON750	ON751	T1 phage genome
T1_2	9827	ON752	ON753	T1 phage genome
T1_3	9710	ON754	ON755	T1 phage genome
T1_4	9958	ON756	ON757	T1 phage genome
T1_5	9782	ON758	ON759	T1 phage genome
cos(T3)	317	ON760	ON761	λ cI857 genome
T3_1	9439	ON762	ON763	T3 phage genome
T3_2	9493	ON764	ON765	T3 phage genome
T3_3	9459	ON766	ON767	T3 phage genome
T3_4	10018	ON768	ON769	T3 phage genome
cos(T7)	317	ON770	ON771	λ cI857 genome
T7_1	10203	ON772	ON773	T7 phage genome
T7_2	9864	ON774	ON775	T7 phage genome
T7_3	10114	ON776	ON777	T7 phage genome
T7_4	9797	ON778	ON779	T7 phage genome
cos(φ80)	317	ON780	ON781	λ cI857 genome
φ80_1	9099	ON782	ON783	φ80 genome
φ80_2	9288	ON784	ON785	φ80 genome
φ80_3	9362	ON786	ON787	φ80 genome
φ80_4	9595	ON788	ON789	φ80 genome
φ80_5	9044	ON790	ON791	φ80 genome
pBR322(cos)	4248	ON593	ON594	pBR322
cos(pBR322)	308	ON597	ON598	λ cI857 genome
pBRcos(48k)	2819	ON705	ON706	pBR322-cos
P1_1	13139	ON707	ON651	P1 phage genome
P1_2	13532	ON652	ON653	P1 phage genome
cat fragment	1116	ON550	ON549	pKD3
P1_3	9115	ON654	ON655	P1 phage genome

P1_4	8520	ON656	ON708	P1 phage genome
λ(4.6k)	4571	ON476	ON477	λ cI857 genome
λ(0.3k)	346	ON456	ON457	λ cI857 genome
λ(4.8k)	4750	ON458	ON459	λ cI857 genome
P1(3.3k)	3342	ON460	ON461	P1 phage genome
P1(3.0k)	2937	ON462	ON463	P1 phage genome
P1(6.0k)	6047	ON464	ON465	P1 phage genome
pBRcos(15k)	3043	ON740	ON743	pBR322-cos
λ12k	12201	ON736	ON737	λ cI857 genome
pBRcos(20k)	3043	ON741	ON743	pBR322-cos
λ17k_A (= λ22k_A)	7091	ON736	ON178	λ cI857 genome
λ17k_B	10057	ON179	ON738	λ cI857 genome
pBRcos(25k)	3043	ON742	ON743	pBR322-cos
λ22k_B	9773	ON179	ON180	λ cI857 genome
λ22k_C	5213	ON709	ON739	λ cI857 genome
T1(+cos) check	441 or 746	ON968	ON969	T1 or T1(+cos) phage genome

Table S3: Combination of PCR fragments

Name	Fragment 1	Fragment 2	Fragment 3	Fragment 4	Fragment 5	Fragment 6
**λ phage**	λ_1	λ_2	λ_3	λ_4	λ_5	
**λ(Δea47)**	λ_1	λ_2	λ_3(Δea47)A	λ_3(Δea47)B	λ_4	λ_5
**λ(Δea31-ea59)**	λ_1	λ_2	λ_3(Δea31-ea59)A	λ_3(Δea31-ea59)B	λ_4	λ_5
**λ(Δp35-orf61)**	λ_1	λ_2	λ_3(Δp35-orf61)	λ_4(Δp35-orf61)B	λ_5	
**λ(Δorf61-gam)**	λ_1	λ_2	λ_3	λ_4(Δorf61-gam)A	λ_4(Δorf61-gam)B	λ_5
**λ(Δexo)**	λ_1	λ_2	λ_3	λ_4(Δexo)A	λ_4(Δexo)B	λ_5
**λ(Δbet)**	λ_1	λ_2	λ_3	λ_4(Δbet)A	λ_4(Δbet)B	λ_5
**λ(Δkil-sieB)**	λ_1	λ_2	λ_3	λ_4(Δkil-sieB)A	λ_4(Δkil-sieB)B	λ_5
**λ(ΔrexB-cI)**	λ_1	λ_2	λ_3	λ_4(ΔrexB-cI)A	λ_4(ΔrexB-cI)B	λ_5

**λ(Δren-ninI)**	λ_1	λ_2	λ_3	λ_4	λ_5(Δren-ninI)A	λ_5(Δren-ninI)B
**λ(Δbor-p79)**	λ_1(Δbor-p79)	λ_2	λ_3	λ_4	λ_5(Δbor-p79)A	
**λ(Δnin Δbor)**	λ_1	λ_2	λ_3	λ_4	λ_5(Δren-ninI)A	λ_5(Δnin Δbor)B
**λ(Δkil ΔcI Δnin Δbor)**	λ_1	λ_2	λ_3	λ_4(Δkil, ΔcI)	λ_5(ΔcI Δnin Δbor)	
**T1(+cos)**	cos(T1)	T1_1	T1_2	T1_3	T1_4	T1_5
**T3(+cos)**	cos(T3)	T3_1	T3_2	T3_3	T3_4	
**T7(+cos)**	cos(T7)	T7_1	T7_2	T7_3	T7_4	
**φ80(+cos)**	cos(φ80)	φ80_1	φ80_2	φ80_3	φ80_4	φ80_5
**pBRcos-48k**	pBRcos(48k)	P1_1	P1_2	cat fragment	P1_3	P1_4
**pBRcos-15k**	pBRcos(15k)	λ12k				
**pBRcos-20k**	pBRcos(20k)	λ17k_A	λ17k_B			
**pBRcos-25k**	pBRcos(25k)	λ22k_A	λ22k_B	λ22k_C		
**22k for Exo III assembly (for Fig. S1)**	λ(4.6k)	λ(0.3k)	λ(4.8k)	P1(3.3k)	P1(3.0k)	P1(6.0k)

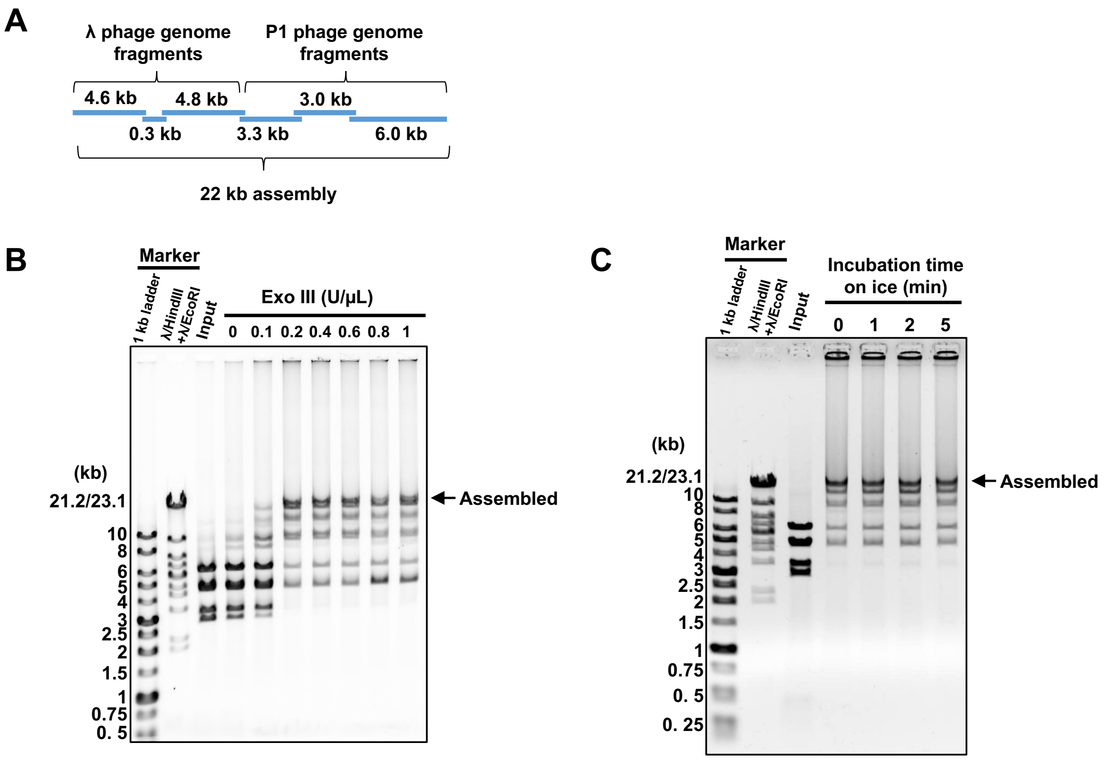

Figure S1. Examination of DNA assembly conditions by using Exo III

A. DNA fragments used for examination of assembly conditions. PCR fragments of the indicated sizes prepared from the λ phage genome and the P1 phage genome as templates were used for Exo III assembly. Adjacent fragments overlap by 50 bp each.

B. DNA assembly according to the concentration of Exo III. Exo III assembly was carried out as describe in the meterials and methods section, except that the concentration of Exo III was changed from 0 to 1 mU as indicated. After assembly reaction, the assembled DNA was analyzed by 0.7% agarose gel electrophoresis. Arrow indicates the assembled DNA.

C. DNA assembly according to the time of incubation on ice after the addition of DNA to the Exo III assembly solution with the Exo III concentration of 0.6 U/μL. DNA was incubated on ice for the indicated time before transferring to 75°C. The assembled DNA was analyzed by 0.7% agarose gel electrophoresis. Arrow indicates the assembled DNA.

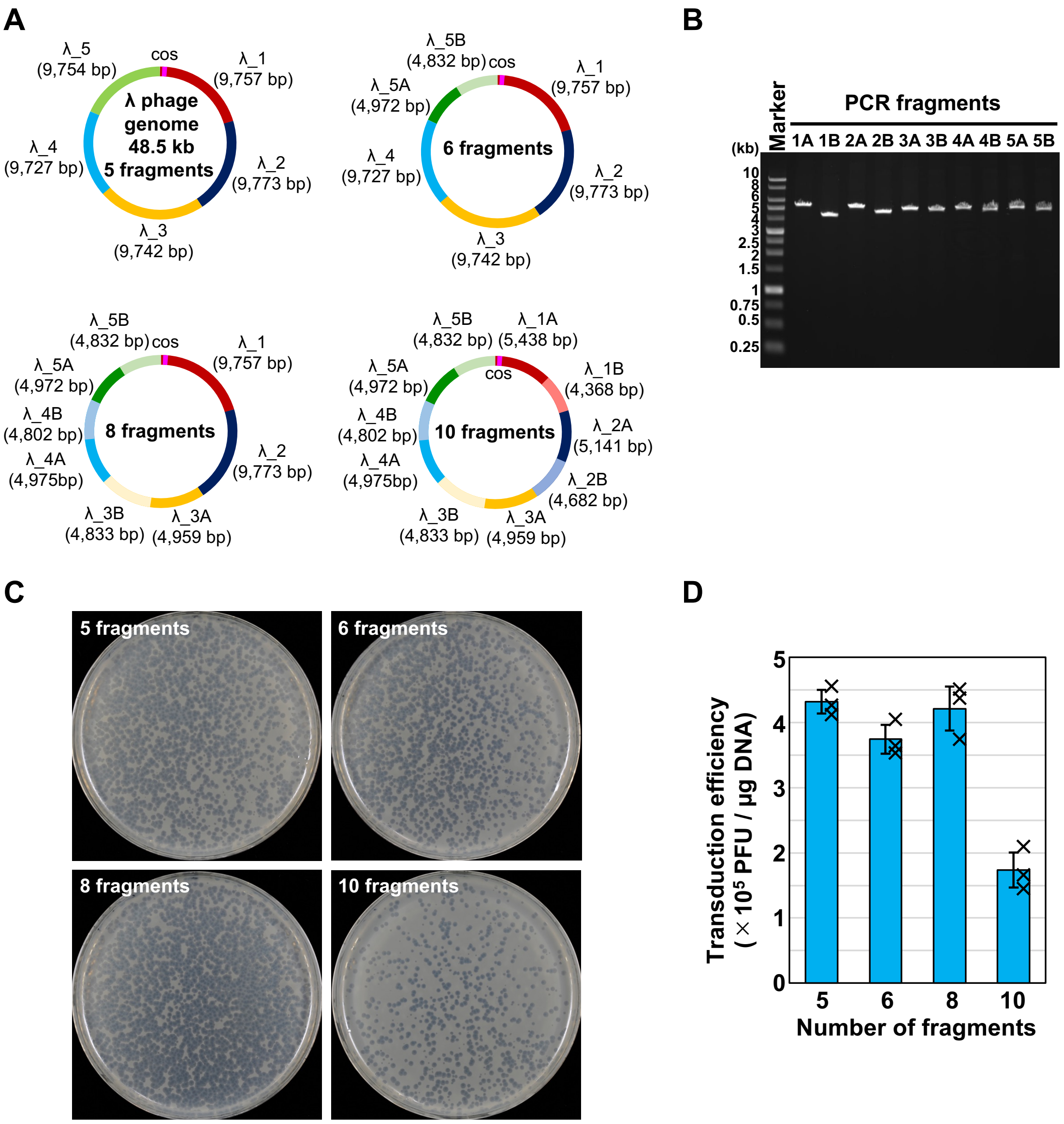

Figure S2. Efficiency of iPac by number of fragments assembled

A. Design of 5, 6, 8 and 10-fragment assembly. In 10-fragment assembly, each of the fragment used for 5-fragment assembly was split into two fragments of about 5 kb.

B. Prepared PCR fragments were analyzed by 0.7% agarose gel electrophoresis.

C. Plaque formation after assembling the indicated number of fragments by iPac.

D. Efficiency of iPac according to the number of fragments. Averages of three biological replicates are shown. Error bars indicate the standard deviation and crosses indicate the actual values.

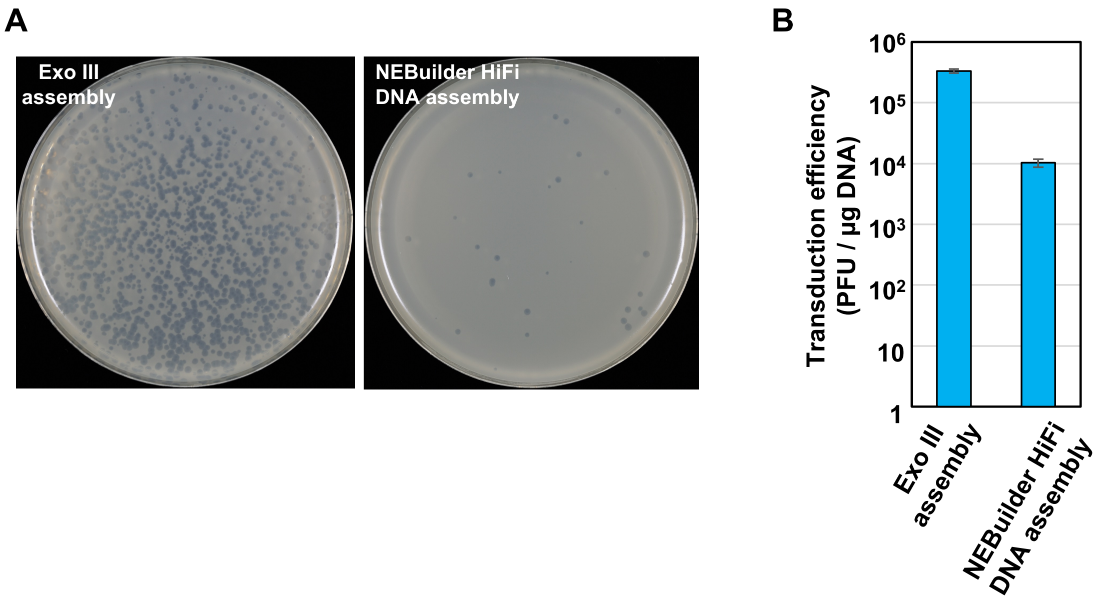

Figure S3. iPac with NEBuilder HiFi DNA assembly

A. Plaque formation of λ phage constructed from five PCR fragments by iPac using Exo III assembly and NEBuilder Hifi assembly kit.

B. Transduction efficiency by iPac using Exo III assembly and NEBuilder DNA assembly kit.

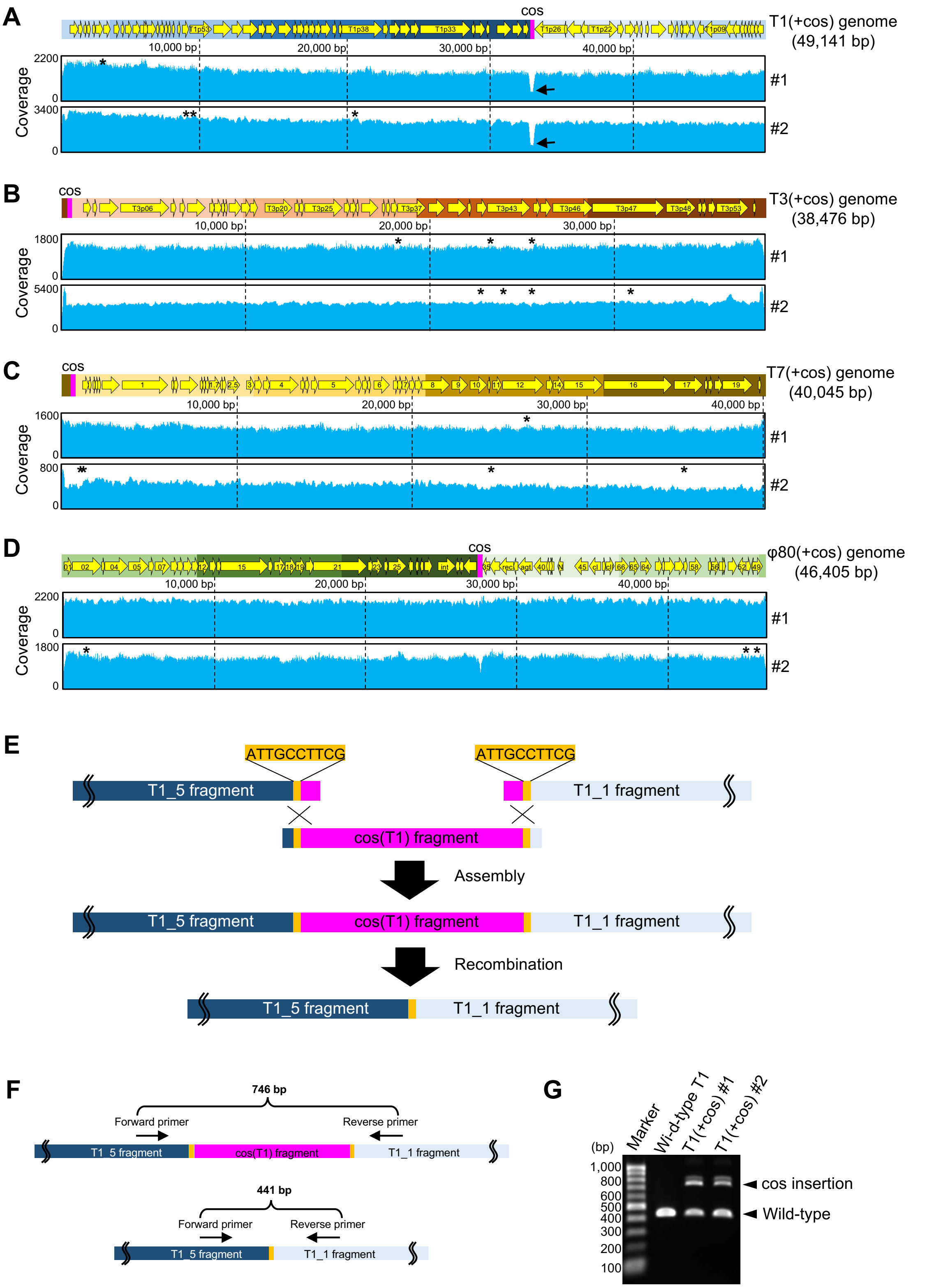

Figure S4. NGS analysis of various phages constructed by iPac.

A - D. Genomes of constructed T1 phage (A), T3 phage (B), T7 phage (C) and φ80 (D) were analyzed by NGS. The reads obtained by NGS were mapped to the reference genome sequences. Asterisks

indicate single nucleotide substitution mutations. Arrows indicate the drop of reads in the inserted cos region.

E. Expected mechanism of cos deletion in T1 phage construction. 10 bp homologous sequences that occurred during the fragments design step are indicated in orange and cos fragment in magenta.

F. Scheme of confirmation of the inserted cos region of the T1(+cos) phage genome. Primers for PCR were designed to amplify the region flanking the inserted cos site.

G. PCR products amplified using wild-type T1 or T1(+cos) phage genome as templates were analyzed by 1.5% agarose gel electrophoresis. The wild-type T1 phage genome without cos insertion was used as a negative control.

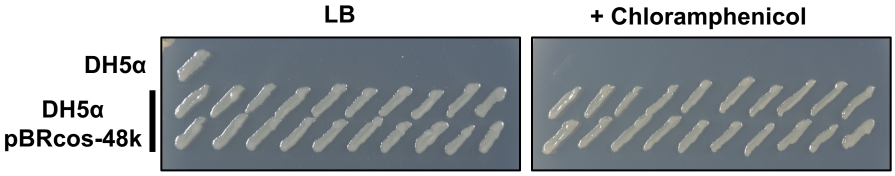

Figure S5. Confirmation of chloramphenicol resistance of *E.coli* DH5α strain harboring pBRcos-48k constructed by iPac.

Twenty Randomly selected colonies of DH5α pBRcos-48k constructed by iPac were streaked onto LB plate with or without 15 μg/mL chloramphenicol and confirmed the chloramphenicol resistance. DH5α without the plasmid was used as a negative control.

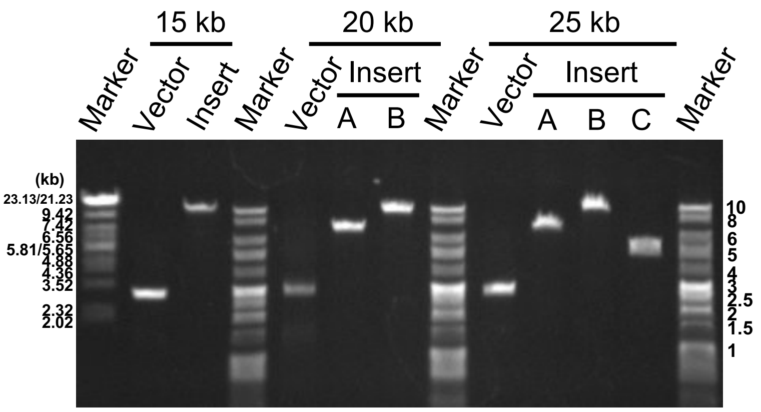

Figure S6. PCR products prepared for the assembly of 15, 20 25 kb plasmids by iPac. PCR products were analyzed by 0.7% agarose gel electrophoresis.

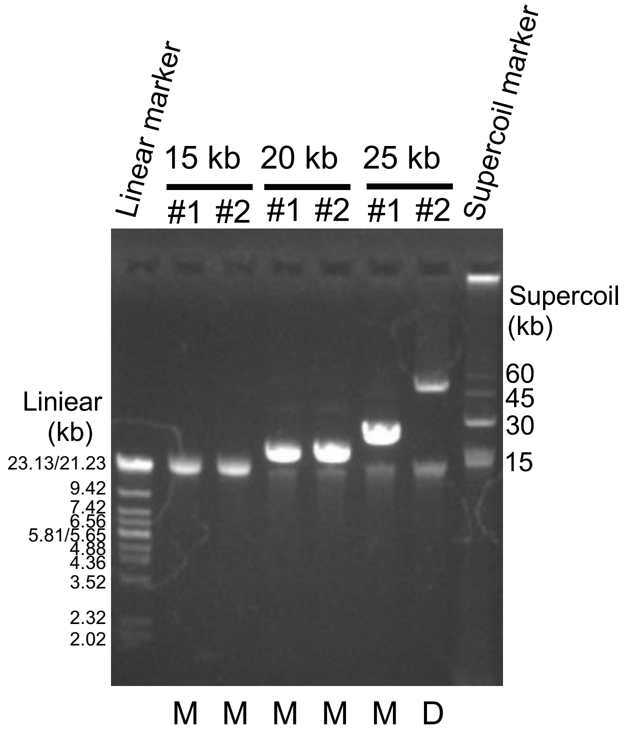

Figure S7. 15, 20 and 25 kb plasmids recovered after introduction into DH5α.
Plasmids recovered from DH5α was analyzed by 0.7% agarose gel electrophoresis. “M” indicates monomer and “D” indicates dimer.

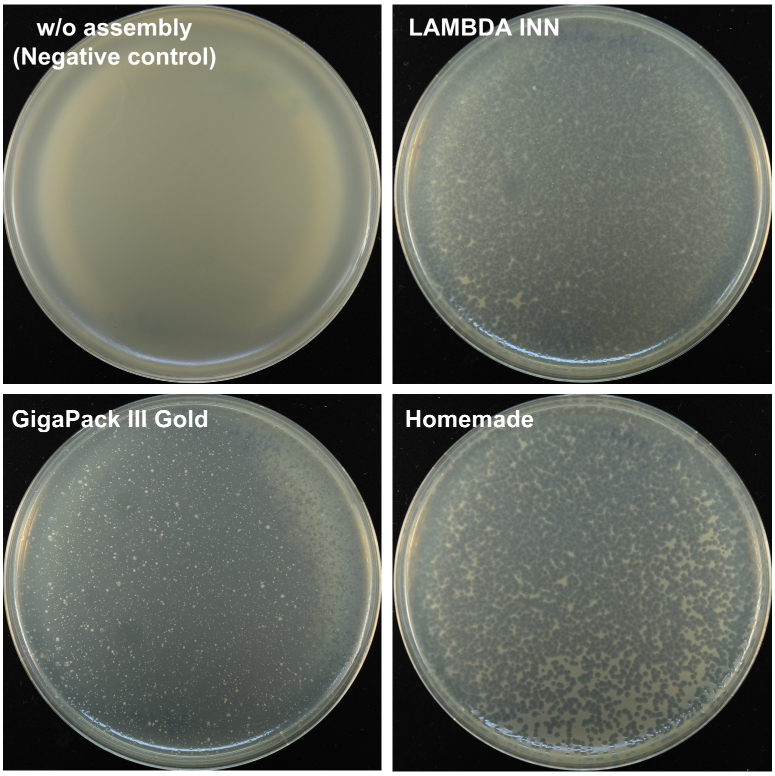

Figure S8. iPac with commercially available and homemade packaging extracts.

iPac with Exo III assembly was carried out using LAMBDA INN in vitro Packaging Kit (Nippon Gene), Gigapack III Gold Packaging Extract (Agilent) and homemade packaging extract, and formation of plaques was observed. A plate prepared without assembly of DNA is shown as negative control.
